# Novel insights into the quality changes and metabolite transfer rules of pickles during fermentation: Pickle versus pickle solution

**DOI:** 10.1016/j.fochx.2025.102203

**Published:** 2025-01-24

**Authors:** Yanan Xia, Wenjing Zhu, Yufan Su, Yongfu Chen

**Affiliations:** College of Food Science and Engineering, Inner Mongolia Agricultural University, Hohhot 010018, China

**Keywords:** Low salt, Pickled vegetables, Fermentation process, Texture, Flavor, Metabolite

## Abstract

The key metabolites and control targets involved during fermentation of pickle need to be identified to improve its quality. This study aims to explore the changes in the quality of white radish during fermentation and the formation rules of key and harmful metabolites in pickle and pickle solutions. Results showed that the flavor profile of the pickle became more complex during fermentation. After fermentation of the pickle solution, metabolites such as D-tartaric acid and mannitol were significantly up-regulated, and the metabolism of nucleotides, histidine and riboflavin was active. Additionally, metabolites in pickle such as ginnalin B and Gly-Asn-Phe were notably up-regulated, and nucleotide metabolism and phenylalanine metabolism were active. 32 metabolites from the pickle were found to dissolve into the pickle solution, and 18 metabolites in the pickle solution penetrated into the pickle, in order to provide the theoretical basis for the quality control and industrial development of low-salt pickle.

## Highlights

• In pickle and solution fermentation, metabolites and pathways were different.
• 32 metabolites from the pickle were found to dissolve into the pickle solution.
• 18 metabolites in the pickle solution penetrated into the pickle.

## Graphical abstract

**Figure:**
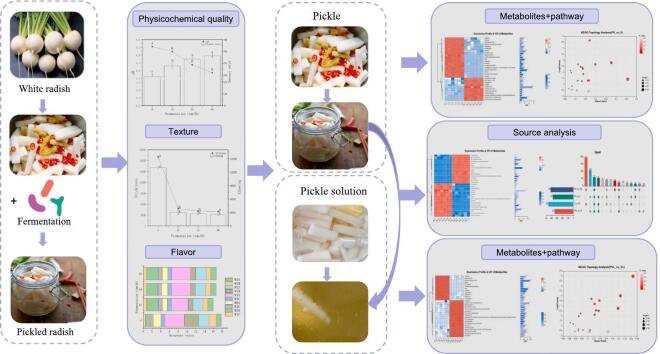

## 1 Introduction

Kimchi represents a traditional form of pickled food, crafted from fresh vegetables, with a history spanning over two millennia. The kimchi-making fermentation process encompasses submerging vegetables in a soaking solution to enable lactic acid fermentation. Chinese, Japanese, and Korean kimchi varieties are globally acknowledged as health-promoting vegetable-fermented products. Their production conditions can be considered as benchmarks and indicative of the development trends for vegetable-pickled products (Li, 2006). Considering the limitations of pickled vegetables, such as their high salt content, high nitrate content and easy spoilage, Japan proposed low salinisation of food as early as the 1970s, successfully developed low-salinisation technology for pickled vegetables and proposed a new nutritional standard for the first time in the 1980s, which reduced salt intake (Khan et al., 2024).

In China, numerous regions are engaged in vegetable pickling. The climatic conditions in the northern part of the country are highly conducive to lactic acid fermentation. Moreover, the abundance of vegetable resources has spurred the robust development of the pickle industry. Notwithstanding the continuous production of pickled vegetables, the production technology has remained relatively underdeveloped. A substantial portion of the industry is composed of small and micro-enterprises, frequently family-owned or operating out of small workshops. The majority of manufacturers still rely on the traditional natural fermentation process for pickled vegetable production. Product quality is predominantly regulated by the personal experience of employees. This approach leads to low production efficiency of pickled vegetables and subpar product quality (Chen et al., 2024).

The fermentation process is pivotal in controlling the quality of low-salt pickled vegetables. An ever-growing number of researchers have delved into the quality changes of kimchi during fermentation. Lu et al. (2007) incorporated *Lactobacillus plantarum* B4 and *Lactobacillus curvatus* A8 into the production process of radish pickles. They discovered that, regardless of the addition of a starter culture, a significant impact was exerted on the viable bacteria count and lactic acid content within the pickles. Yan et al. (2008) employed different fermentation methods to ferment cabbage. Through the analysis of nitrite and microorganism quantity changes, along with product sensory evaluation, they found that the quality of inoculated-fermented cabbage was superior. By utilizing low-salt curing and processing techniques, diverse raw products of pickled vegetables can be obtained. In line with the requirements for the storage, edible safety, and nutritional quality of preserved vegetables, the quality of pickled vegetables can be comprehensively evaluated in terms of their pH value, total acid content, lactic acid bacteria count, reducing sugar content, organic acid content, and the texture of pickled vegetables (Liang et al., 2016; Ye et al., 2022).

The pickling solution, comprising salt, sugar, vinegar, and spices, significantly impacts pickle quality. Salt, via high osmotic pressure, controls microbial growth, affects texture, and promotes flavor formation. Sugar modulates taste and contributes to quality through osmotic-related effects. Vinegar, by lowering pH, inhibits microbes, preserves color, and stabilizes enzymes. Spices add unique flavors and some have antibacterial properties, reducing nitrite. The concentration of the pickling solution is closely linked to the quality of pickles. High-concentration solutions provide strong anti-corrosion but can cause issues like dehydration, over-salting, and flavor inhibition. Low-concentration ones reduce saltiness and nutrient loss but are more prone to spoilage and affect texture and flavor. The pH of the pickling solution is crucial. Acidic conditions favor beneficial fermentation and color/flavor development, while alkaline ones, despite stabilizing pigments, may lead to poor taste. At neutral pH, microbial balance is maintained, but other factors must be considered for quality control due to its limited impact on color and flavor.

China ranks among the leading producers of white radishes, a significant portion of which is channeled into the production of pickled white radishes. These pickled radishes exhibit distinctive quality attributes. Their flavor profile is rich and diverse, offering a delightful combination of tastes, while their texture is crisp, tender, and refreshing. Nutritionally, not only do they retain the inherent nutrients of white radishes, but the fermentation process also introduces beneficial components, promoting intestinal health. As a result, pickled white radishes enjoy substantial market demand. Nevertheless, challenges such as the propensity for yellowing during pickling persist.

Low-salt fermentation of pickled vegetables is an inevitable trend in the pickled vegetable industry, with the quality of the final products being closely related to the quality control of the fermentation process. However, the key metabolites and control targets involved in the fermentation of low-salt fermented pickled vegetables remain unclear. This study aims to explore the changes in physical, chemical, texture, flavor, metabolites and other qualities of low-salt fermented white radish during the dry fermentation process and investigate the formation rules of key and harmful metabolites in pickle and pickle solution during the fermentation process (Graphic abstract) to provide a theoretical basis for quality control and industrial development of pickled vegetables during low-salt fermentation.

## 2 Materials and methods

### 2.1 Experimental raw materials

The starter culture consisting of a 1:2 ratio of *Pediococcus acidilactici* S3–3 and *Lactobacillus delbrueckii* QS306 was obtained from the laboratory of Ethnic Food Research Team of Inner Mongolia Agricultural University (Hohhot, China). *Pediococcus acidilactici* S3–3(Sc9–7), which was isolated from Inner Mongolia Sauerkraut, had strong acid - production and fermentation abilities. It also exhibited strong antibacterial capacity, and the diameters of the antibacterial zones against *Escherichia coli, Bacillus cereus*, and *Micrococcus luteus* could reach 15.0 ± 1.0, 19.0 ± 1.0, and 19.7 ± 0.6 mm respectively (Li et al., 2012). *Lactobacillus delbrueckii* QS306 (YN-01), isolated from Inner Mongolia fermented milk, had good aroma-production characteristics and its fermentation broth had a high ACE inhibition rate (Shuang et al., 2015).

The raw materials used for the preparation of pickled vegetables, including white radish, edible salt, white sugar, Niulanshan Erguotou liquor, ginger and garlic were procured from a supermarket located in Hohhot, Inner Mongolia.

### 2.2 Preparation of pickled vegetables

White radish was thoroughly washed, peeled and cut into small pieces (1 cm*1 cm*1 cm). A 6 % salt solution was added, and the mixture was mixed thoroughly. Excess water was discarded, and the radish was blanched in water at 90 °C for 3 min. After cooling at 25 °C for 20 min, additives such as 5 % sucrose, 3 % garlic, 2 % ginger and 1 % liquor were added, obtaining the sample of vegetable and vegetable solution. Then a starter culture (viable bacteria count 1.5 × 10^8^ CFU) was inoculated. Distilled water was added in a sufficient quantity until the radish was fully submerged. Finally, the sample was sealed with plastic wrap and fermented in an incubator at 27 °C for 24 h. Samples were collected at 12 h intervals to investigate the quality changes of low-salt pickled vegetables during the fermentation process. The final pickle and pickle solution were also collected to analyze the source and target of beneficial and harmful substances.

### 2.3 Flavor determination

The electronic nose was used to detect the flavor components of pickled vegetables. The chopped white radish sample was placed in a 50 mL centrifuge tube and sealed with plastic wrap. The sample was heated in a 60 °C water bath so that the flavor compounds gather to the top, and then an automatic injector was inserted to extract them. Parameters were set as follows: sampling interval of 1 s, flushing time of 40s, detection time of 120 s, chamber flow of 400 mL/min, initial injection flow of 400 mL/min. Each sensor generated a respective response value for specific substance, and the corresponding relationship was depicted in Table S1.

### 2.4 Quality evaluation

During the production of pickled vegetables, a sensory evaluation panel consisting of 10 members was established to perform comprehensive scoring of low - salt fermented pickled vegetables from four aspects: color, flavor, taste, and overall palatability. The average score of these 10 panelists was then calculated. The detailed scoring criteria are presented in Table S2. A Konica Minolta CR-20 portable color difference meter (Guangzhou Chuoharmonic Instrument Equipment Co., Ltd.) was employed. Prior to measurement, the instrument was calibrated according to the manufacturer's instructions using a standard white calibration plate. The surface of the pickled vegetable samples was carefully cleaned to remove any debris or moisture. The color difference meter was then gently placed on the sample surface, ensuring uniform contact. The surface brightness value (L*), redness value (a*), and yellowness value (b*) were recorded. Subsequently, the Chromatic Aberration (ΔE) was calculated using the formula: ΔE = [(ΔL*)^2^ + (Δa*)^2^ + (Δb*)^2^]^(1/2), where ΔL*, Δa*, and Δb* represent the differences in L*, a*, and b* values between the sample and a reference.

The nitrite content in pickles was determined via ultraviolet spectrophotometry. A stock nitrite solution (sodium nitrite, AR grade) was diluted with deionized water to prepare standard solutions of 0, 5, 10, 15, 20, 25 μg/mL. A 5.0-g pickled vegetable sample was homogenized with 50 mL of deionized water in a blender. The homogenate was filtered through Whatman No. 42 filter paper. To 10 mL of the filtrate, 2 mL of 10 % (*w*/*v*) ammonium sulfamate was added to eliminate nitrate interference. After 5 min, 2 mL of 0.4 % (w/v) N-(1-naphthyl)-ethylenediamine dihydrochloride and 2 mL of 2 % (w/v) sulfanilic acid in 30 % (*v*/v) hydrochloric acid were added sequentially. The mixture was left at room temperature (25 ± 2 °C) for 15 min for color development. Absorbance of standard and sample solutions was measured at 538 nm using a UV–visible spectrophotometer, zero-adjusted with a blank (all reagents minus nitrite). The sample's nitrite content was determined by interpolation from the standard curve of absorbance values of the standard solutions.

### 2.5 Metabolite detection

In a 2-ml centrifuge tube, 50 mg of the sample was precisely measured and a 6-mm diameter grinding bead was added. Then, 400 μL of an extract solution (methanol:water = 4:1 (*v*/v)) containing 0.02 mg/mL of the internal standard (L-2-chlorophenylalanine) was introduced for metabolite extraction. The sample solution was ground with a frozen tissue grinder for 6 min at −10 °C and 50 Hz, followed by low-temperature ultrasonic extraction for 30 min at 5 °C and 40 kHz. The samples were placed at −20 °C for 30 min and then centrifuged for 15 min at 4 °C and 13,000 ×g. The resulting supernatant was transferred to an injection vial with internal intubation for machine analysis.

An ultra-high performance liquid chromatography–tandem Fourier transform mass spectrometry UHPLC-Q Exactive system (Thermo Fisher, USA) was used to detect the analytes. A 3-μL sample was separated on a HSS T3 column (100 mm × 2.1 mm i.d., 1.8 μm) prior to mass spectrometry. Mobile phase A consisted of 95 % water and 5 % acetonitrile, both containing 0.1 % formic acid. Mobile phase B was composed of 47.5 % acetonitrile, 47.5 % isopropanol, and 5 % water, also with 0.1 % formic acid. For the positive-ion mode, the separation gradient was as follows: from 0 to 3 min, mobile phase B increased from 0 % to 20 %; from 3 to 4.5 min, it rose from 20 % to 35 %; from 4.5 to 5 min, it increased from 35 % to 100 % and was held at 100 % from 5 to 6.3 min; from 6.3 to 6.4 min, it decreased from 100 % to 0 % and was maintained at 0 % from 6.4 to 8 min. For the negative - ion mode, the gradient was: from 0 to 1.5 min, mobile phase B increased from 0 % to 5 %; from 1.5 to 2 min, it rose from 5 % to 10 %; from 2 to 4.5 min, it increased from 10 % to 30 %; from 4.5 to 5 min, it increased from 30 % to 100 % and was held at 100 % from 5 to 6.3 min; from 6.3 to 6.4 min, it decreased from 100 % to 0 % and was kept at 0 % from 6.4 to 8 min. The flow rate was set at 0.40 mL/min, and the column temperature was maintained at 40 °C. Each sample group was measured in triplicate (Zhao et al., 2018).

The raw LC-MS data were processed in Progenesis QI (Waters Corporation, Milford, USA), a metabolomics software. The operations included baseline filtering, peak identification, integration, retention time correction, and peak alignment, generating a data matrix of retention time, mass-to-charge ratio, and peak intensity. Concurrently, MS and MSMS data were matched with public metabolomics databases (HMDB: http://www.hmdb.ca/ and Metlin: https://metlin.scripps.edu/) and a self - built database to obtain detailed metabolite information (Yu & Xu, 2007). Post-database search, the data matrix was uploaded to cloud.majorbio.com for further analysis.

The data matrix first underwent pre-processing steps. The 80 % rule was used to eliminate missing values, retaining variables with non-zero values in at least 80 % of samples in one group. Missing values were filled with the minimum value in the original matrix. The sum normalisation method was applied to normalise the response intensity of essential spectrum peaks, and variables with a relative standard deviation >30 % in QC samples were removed. Log10 transformation was then performed to get the final data matrix for subsequent analysis. Next, the ropls package in R (Version 1.6.2) was used for PCA and OPLS-DA on the pre-processed data. Seven repeated interactive verifications were carried out to assess the model's stability and accuracy. Metabolites with significant differences were selected based on VIP > 1 from the OPLS-DA model and *p*-value <0.05 from the *t*-test. The KEGG database was used to analyze metabolite differences, and the Python package scipy.stats was employed for pathway enrichment analysis, with Fisher's exact test identifying the most relevant biological pathways to the experimental treatment.

### 2.6 Statistical analysis of data

Microsoft Excel and SPSS 27.0 software were used to process the experimental data, and Origin 2018 mapping was performed. All experiments were performed in parallel three times. The results presented in the figures are expressed as mean ± standard error, and different letters in the same column indicate significant differences (*p* < 0.05). The same letters in the same column indicated no significant differences (*p* > 0.05).

## 3 Results and discussion

### 3.1 Changes in the physical and chemical qualities of pickles

**Fig. 1:**
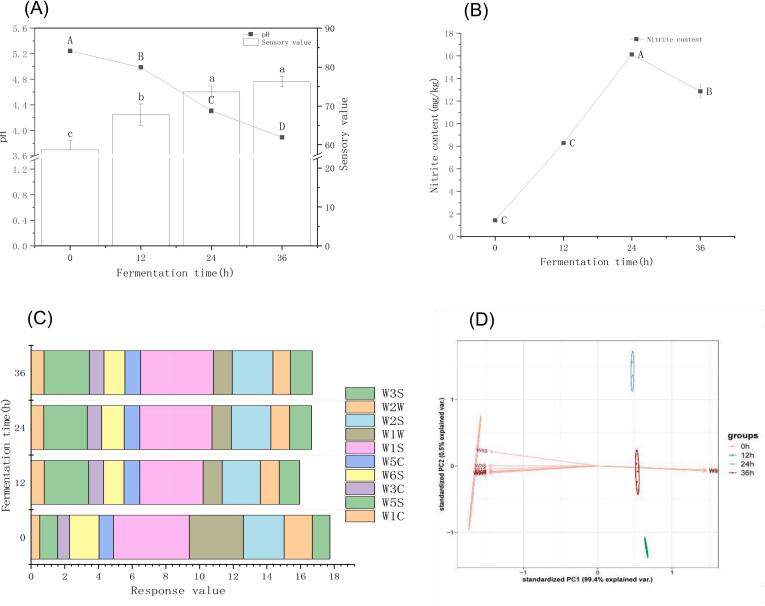
Quality change of pickles during fermentation. (A) pH and sensory scores. (B) Nitrite content. (C—D) Flavor.

Appropriate acidity played an important role in the quality evaluation of pickled dishes. Fig. 1A demonstrated that the pH values of pickled vegetables during the low-salt fermentation process exhibited a significant variation (*p* < 0.05). The pH value of pickled vegetables fermented for 0 h was the highest, reaching 5.28 ± 0.01. During fermentation, the pH of the pickled vegetables gradually decreased. The pH values of the 12 - and 24 - h fermentation groups were recorded as 4.98 ± 0.01 and 4.31 ± 0.01, respectively. The pH of pickled vegetables fermented for 36 h was the lowest, measuring 3.91 ± 0.04. These findings indicated that as the fermentation time increased, the strain activity was enhanced and the acid-production rate was accelerated, resulting in a faster pH decline. Ye et al. (2022) discovered that during the fermentation process of pickled radish, lettuce, and cowpea, the pH dropped below 4.0 and the total acid content was significant (*p* < 0.05), which was consistent with the results of this study. At the same time, the sensory scores of pickled vegetables gradually increased during the fermentation process and remained stable until 36 h after fermentation, indicating that the fermentation process promoted the overall quality of pickled vegetables.

A significant change occurred in the nitrite content of low-salt fermented pickled vegetables during fermentation (p < 0.05, Fig. 1B). Initially, at 0 h of fermentation, the nitrite content of pickled vegetables was 1.45 ± 0.01 mg/kg. As the fermentation time progressed, the nitrite content of pickled vegetables first increased and then decreased. Specifically, the nitrite content reached its peak at 24 h, amounting to 16.12 ± 0.16 mg/kg. The increase in nitrite content could be attributed to the fact that, when pickles began the curing process, the environment within the container was favorable for the proliferation of certain bacteria, including nitrate - reducing bacteria. These bacteria were capable of promoting the reduction of nitrate to nitrite. However, with the continuation of fermentation, the nitrite content started to decline. In the 36 - h fermentation group, the nitrite content was recorded as 12.85 ± 0.59 mg/kg. This phenomenon was due to the proliferation of lactic acid bacteria as the curing time increased. The lactic acid bacteria inhibited the growth and reproduction of nitrate - reducing bacteria, thereby leading to a decrease in nitrite content. Notably, the nitrite content of the final product met the national standard of 20 mg/kg.

As presented in Table 1, significant differences in L* (brightness) values were detected during the fermentation of low-salt pickled vegetables (*p* < 0.05). The L* value of pickles at 0 h of fermentation was the highest, attaining 67.03 ± 0.67. Nevertheless, as the fermentation advanced, the L* value started to decline. The L* value of pickled vegetables fermented for 36 h was the lowest, measured at 63.70 ± 0.53. Regarding the a* value of pickled vegetables during low - salt fermentation, it first increased and then decreased throughout the fermentation process. The a* value of pickled vegetables fermented for 24 h reached its peak, with a value of 1.00 ± 0.15, suggesting that the pickled vegetables had a tendency to turn red during the mid - fermentation stage. In contrast, the b* value of pickled vegetables during low - salt fermentation demonstrated an upward trend, mainly due to the formation of yellow pigments. Significantly, the b* value of pickled vegetables fermented for 36 h was the highest, reaching 7.27 ± 0.26. At this point, with △E (chromatic aberration) > 2, it indicated that the final fermented product had a visible color difference compared to the unfermented raw material. Ou et al. (2022) discovered that during the fermentation of radish pickles, the L* value showed a decreasing tendency, while no significant differences were found in the a* and b* values. These results were consistent with the color trend observed in the present study.

**Table 1:** Chroma changes of of pickles during fermentation.

Chroma	Fermentation time/h			
	0	12	24	36
L*	67.03 ± 0.67^a^	66.20 ± 2.1^bc^	65.83 ± 0.71^b^	63.70 ± 0.53^c^
a*	0.60 ± 0.00^b^	0.70 ± 0.06^b^	1.00 ± 0.15^a^	0.43 ± 0.03^b^
b*	2.53 ± 0.34^c^	3.20 ± 0.06^c^	5.13 ± 0.94^b^	7.27 ± 0.26^a^
△E	–	1.07 ± 0.14	1.99 ± 0.46	3.07 ± 0.32

### 3.2 Flavor changes of pickles during fermentation

The changes in flavor during the fermentation of low-salt pickled vegetables were presented in Fig. 1C. As illustrated in Fig. 1C, the flavor substances of the raw materials at 0 h of fermentation were predominantly concentrated in organic components such as methane, hydrocarbons, and sulfur, as well as nitrification components and hydrogen. As the fermentation advanced, the organic and nitrification components of hydrocarbons and sulfur gradually diminished, while the ammonia - oxide compounds gradually increased. The flavors of pickled vegetables fermented for 24 h and 36 h were similar, and the flavor substances of the fermented pickled vegetables mainly consisted of flavor-related compounds and ammonia oxides.

Principal component analysis (PCA) of the sample flavor was conducted during fermentation. As depicted in Fig. 1D, PC1 accounted for 99.4 % and PC2 accounted for 0.5 %, suggesting that these two principal components could represent the main characteristics of the sample's volatile flavor. During the fermentation process, the samples within each group were spatially separated from one another. This separation implies that the samples in each group manifested discernible differences in flavor, enabling their differentiation. The PCA and loading diagram (Fig. 1D) revealed that the flavor of the 0 h sample was mainly attributed to flavor-related substances and alkanes. During the fermentation process, the flavors of the 36 h samples consisted of ammonia oxides, alkanes, alcohols, and organic sulfides. After fermentation, the flavor composition of the samples became more complex. Huang et al. (2021) investigated the volatile flavor substances of Hami melon young fruit pickles and detected a total of 43 substances, among which hydrocarbons and alcohols were the main flavor components. Xue (2023) found that esters and alcohols were the most abundant volatile compounds in the production of low - nitrite pickles, and the contents of ketones, aldehydes, hydrocarbons, acids, and phenols increased with the increase in fermentation time. These results indicated that fermentation significantly improved the flavor composition and richness of pickles.

### 3.3 Comparative analysis of metabolites before and after fermentation

**Fig. 2:**
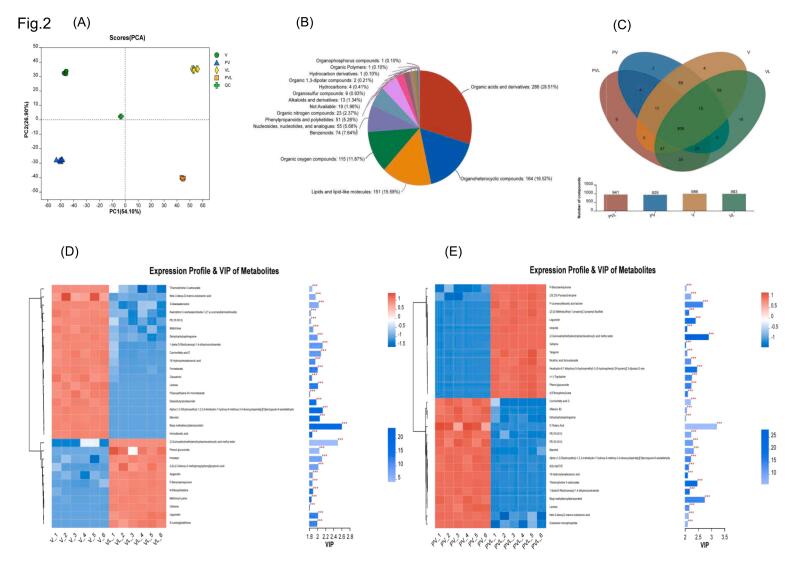
Comparison of metabolites of pickle and pickle solution before and after fermentation. (A) Principal component analysis. (B—C) Pie chart and venn chart of substance composition. (D-E) Differential metabolites of pickles and pickles solution before and after fermentation.

A total of 969 metabolites were identified in the pickle - solution samples using MS1 and MS2 techniques. As presented in Fig. 2B, the identified metabolites consisted of 286 organic acids and their derivatives, 164 organic heterocyclic compounds, 151 lipids and lipid molecules, 115 organic oxygen compounds, 74 phenyl derivatives, 55 nucleosides, nucleotides and analogues, 51 phenylpropyl and polyketones, 23 organic nitrogen compounds, 13 alkaloids and their derivatives, nine organic sulfide compounds, four hydrocarbons, two organic 1,3 - dipole compounds, one hydrocarbon derivative, one organic polymer, and one organophosphorus compound.

In the experiment, PCA was employed to determine the metabolite differences between pickles and pickle solutions before and after fermentation (Fig. 2A). In the cationic mode, the contribution rates of PC1 and PC2 were 54.10 % and 26.90 % respectively, with a combined contribution rate of 81.00 %. In the anionic mode, their contribution rates were 80.10 % respectively, suggesting that PCA could account for the overall information of the sample. Fig. 2A showed that the four groups of samples were located in different quadrants, indicating distinct differences among the four groups. The quality-control samples overlapped, demonstrating that the data in this study were reliable and repeatable.

### 3.4 Metabolic changes in pickle solution before and after fermentation

#### 3.4.1 Differential metabolites of pickle solutions

**Fig. 3:**
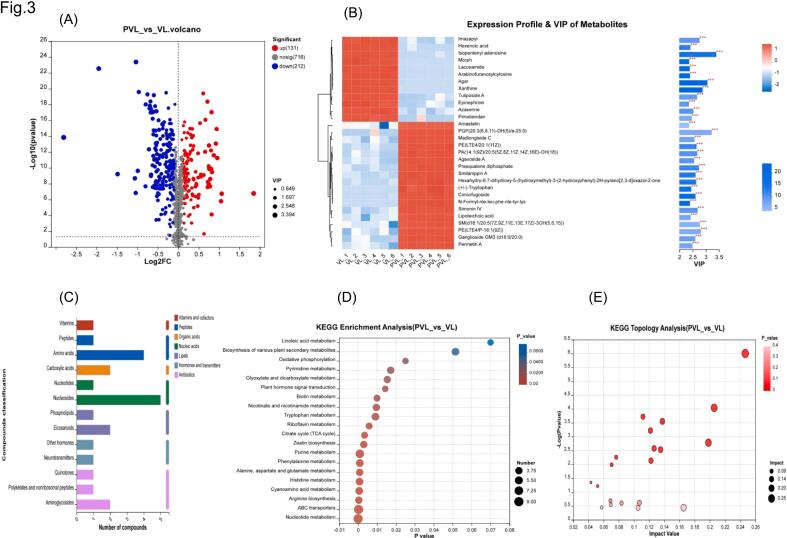
Comparison of metabolites and metabolic pathways before and after fermentation of pickle solution. (A) Volcanic maps. (B) VIP maps of differential metabolites. (C) Differential metabolite composition. (D-E) Enrichment of metabolic pathways during fermentation.

Based on the volcano plot shown in Fig. 3A, 343 differential metabolites were generated before and after the fermentation of the pickle solution (*p* < 0.05, VIP > 1). This set consisted of 131 up - regulated and 212 down - regulated metabolites, which were classified into seven distinct groups (Fig. 3C). The groups included seven nucleic acids, five peptides, four antibiotics, three lipids, two organic acids, two hormones and transmitters, one vitamin and cofactor. Specifically, the key differential metabolites formed post - fermentation of the vegetable solution were analyzed according to VIP values (Fig. 3B). It was found that 12 metabolites, such as xanthine, hexenoic acid, isopentenyl adenosine, and arabinofuranosyl cytosine, were significantly down - regulated. In contrast, D - tartaric acid, mannitol, tryptophan, and 18 other metabolites were significantly up - regulated.

Hexenoic acid, acting as an inhibitor of gamma - aminobutyric acid (GABA) aminotransferase, is highly selective. It inhibits this enzyme, thereby increasing the concentration of GABA in the brain and exerting anti - epileptic effects (Zhao et al., 2024). After fermentation, the content of hexenoic acid in the pickle solution decreased significantly. It was speculated that this reduction was conducive to the increase of GABA, playing a probiotic role. D - Tartaric acid, a natural organic acid with a white crystalline appearance, can be utilized as a sour agent, resolution agent, and pharmaceutical raw material. It has extensive applications in the pharmaceutical industry for the synthesis of various compounds (Dong et al., 2018). After the fermentation process, the concentration of D - tartaric acid in the pickle solution increased significantly. This indicated that fermentation produced a certain amount of D-tartaric acid, which contributed to the formation of a specific flavor and inhibited the propagation of harmful microorganisms.

**Fig. 4:**
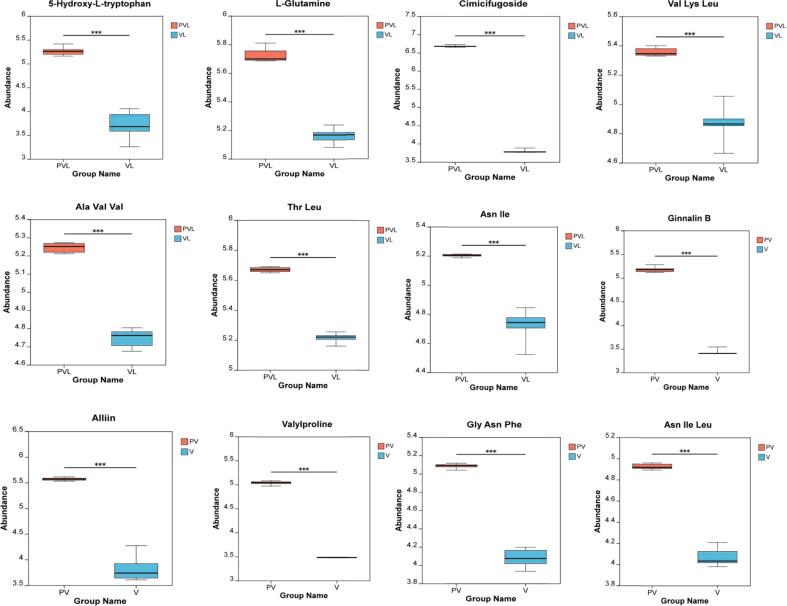
Boxplot of key metabolites in pickle and pickle solution before and after fermentation.

Mannitol, a naturally-occurring polyhydric alcohol, was commonly employed in clinical settings for diuresis, reducing cranial pressure, intraocular pressure, and treating glaucoma. Characterized by a sweet taste, approximately 50 % as sweet as sucrose, it was utilized by dieters and diabetic patients in the food industry (Zheng et al., 2017). Significantly, the concentration of mannitol in the pickled-vegetable solution increased notably after fermentation. This indicated that during fermentation, the synthesis of lactic acid in the glycolysis pathway was impeded. The sour taste of the pickled-vegetable solution remained unchanged, while the sweetness increased slightly in the late fermentation stage. Tryptophan, an essential amino acid in mammals, was involved in numerous physiological processes, such as neuronal function, immunity, and intestinal homeostasis. Tryptophan and its metabolites played a crucial role in health and various diseases, spanning from mental and nervous system disorders to anti-cancer immunity (Claes et al., 1990). Moreover, the metabolite 5-hydroxy-L-tryptophan was found to be significantly up-regulated (Fig. 4). This suggested that the increased concentration of 5-hydroxy-L-tryptophan following the fermentation of the pickled-vegetable solution had an impact on both the flavor and nutritional value of pickled vegetables.

l-Glutamine, the most abundant amino acid in the bloodstream, contributed to the production of glucose, nucleotides, proteins, and glutathione. It served as the primary metabolic fuel for intestinal cells, lymphocytes, macrophages, and fibroblasts (Miller, 1999). The content of L-glutamyl in the pickle solution was significantly up - regulated after fermentation (Fig. 4). This indicated that the concentration of l-glutamine increased following the fermentation of the pickle solution. Such an increase might have contributed to its probiotic effect, thus supporting human health and facilitating recovery. Cimicifugoside belonged to the class of chromogenic ketones. It was extracted from the traditional Chinese medicine windstorm as a monomer, possessing antipyretic, analgesic, anti-inflammatory, anti-platelet aggregation, and anticoagulation properties. It was frequently used in the treatment of certain chronic diseases and those related to the immune system (Xue et al., 2000). Significantly, after fermentation, the concentration of cimicifugoside in the pickle solution was significantly up-regulated (Fig. 4). This suggested that the increased concentration of cimicifugoside in the pickle solution after fermentation could enhance the immune function of the human body and play a probiotic role.

The functional oligopeptides Ala-Val-Val, Val-Lys-Leu, Asn-Ile and Thr-Leu have various biological functions (Fig. 4), such as hormonal action, antithrombotic, antihypertensive, antioxidant, immunomodulatory, cholesterol lowering, antibacterial, viral, and anti-cancer effects. (Zhang & Wang, 2007). Following the fermentation process, the concentration of these short peptides in the pickle solution was significantly up-regulated, indicating that the concentration of short peptides increased after the fermentation of the pickle solution, suggesting a potential enhancement of immune function in the human body and its role as a probiotic agent.

#### 3.4.2 Key metabolic pathways of pickle solution

The Kyoto Encyclopedia of Genes and Genomes (KEGG) database was used to annotate the differential metabolites before and after fermentation to explore the metabolic pathways involved in pickle solution before and after fermentation (Fig. 4D). The top 20 metabolic pathways were selected for analysis. Based on the *P*-value, the most significant metabolic pathways were found to include nucleotide metabolism, ABC transporter, arginine biosynthesis, cyanoamino acid metabolism, histidine metabolism, alanine, aspartate and glutamate metabolism, phenylalanine metabolism and purine metabolism. The pathways with the highest numbers of compounds are nucleotide metabolism and the ABC transporter pathway. Furthermore, a topological analysis (Fig. 3E) identified nucleotide, histidine and riboflavin metabolism as the most significant and important metabolic pathways.

Nucleotide and purine metabolism pathways are common in organisms and fermentation processes. The nucleotide metabolism pathway mainly synthesises and degrades nucleic acids, DNA and RNA, and purine is a member of the nucleotide groups (Yu et al., 2007). During this fermentation process, a total of nine and seven metabolites were involved, including deoxycytidine, hypoxanthine, guanosine monophosphates, adenosine, guanosine, l-glutamine and xanthine, and the other metabolites were significantly down-regulated except for adenosine, guanosine and l-glutamine. The ABC transporter pathway is mainly associated with the nucleotide-binding domain and transmembrane domain and involves nine metabolites, including deoxycytidine, deoxyuridine, betaine, adenosine, l-glutamine, deoxyadenosine and riboflavin (Biemans-Oldehinkel et al., 2006). Within this pathway, adenosine, l-glutamine and guanosine were significantly up-regulated, whereas deoxycytidine, deoxyuridine, betaine, deoxyadenosine and riboflavin were significantly down-regulated.

Adenosine played a pivotal role in cellular energy metabolism, facilitating energy storage and release via the interconversion among ATP, ADP, and adenosine to supply energy for probiotic physiological activities. Guanine, a crucial DNA and RNA base component, was essential for maintaining the stability and normal function of probiotic genetic material. l-glutamine, a multifunctional amino acid, participated in protein and nucleotide synthesis and was significant in cellular antioxidant defense, immune regulation, and acid-base balance (Liu et al., 2023), helping maintain the internal environment stability and normal metabolism in probiotic cells. Together, these three substances enabled probiotics to exert beneficial effects during fermentation, with their stable levels sustaining the basic metabolic processes for pickle flavor formation and ensuring continuous production of specific flavor compounds.

The arginine biosynthesis pathway involves multiple enzymes and metabolic pathways, involving a total of four metabolites, including n-α-acetyl-L-citrulline, l-glutamine, fumaric acid and citrulline (Yan et al., 2015). In this pathway, except for fumaric acid, all substances were significantly up-regulated. N-α-acetyl-L-citrulline served as a crucial provider of the structural unit for the synthesis of arginine (Zhou et al., 2024). l-glutamine, acting as a nitrogen-source donor, not only partook in the synthesis of arginine, but also played a vital role in maintaining the balance of intracellular nitrogen transport and acid-base equilibrium (Kruse et al., 2007). Citrulline, which was a key intermediate in the arginine synthesis pathway, was engaged in the reversal of the urea cycle and might have had implications in cell signaling processes (Morris et al., 2002). Notably, arginine and its precursor metabolites underwent transformation into flavor-active compounds. This conversion significantly influenced the flavor profile of pickles, thereby having a substantial impact on the flavor quality of the pickled products.

Amino acid metabolism is closely associated with the formation of specific flavor substances (Wang, 2007). During the fermentation process, significant enrichment was observed in the cyanoamino acid, histidine, alanine-aspartate-glutamate, and phenylalanine metabolism pathways. The cyanoamino acid metabolic pathway consisted of five metabolites: L-asparagine, prunasine, and lotostalline, all of which were significantly down-regulated. The histidine metabolic pathway included five metabolites: carnosine, L-histidine, imidazolinone-5-propionic acid, and imidazolacrylic acid, all also significantly down-regulated. In the alanine, aspartate, and glutamate metabolic pathways, among the four metabolites, L-asparagine, fumaric acid, and succinic acid were significantly down-regulated, except for l-glutamine. This pathway also involved five metabolites where phenylacetic acid and 2-hydroxycinnamic acid were significantly up - regulated, while fumaric acid, succinic acid, and trans-cinnamic acid were significantly down-regulated. Phenylacetic acid itself possessed an aromatic odor, and its up-regulation during pickle fermentation might have directly added unique aroma components to pickles. 2-hydroxycinnamic acid was a phenolic compound with antioxidant properties. This compound could prevent the oxidative degradation of some flavor substances and maintain the integrity of the pickle flavor.

The metabolic pathway of riboflavin includes its release from food, absorption in the small intestine, intracellular phosphorylation and final excretion and involves a total of three metabolites. Notably, riboflavin reduction was significantly up-regulated while the levels 2,5-amino-6-(5-phospho-D-riboamino) pyrimidine-4(3H)-one riboflavin were significantly down-regulated. Riboflavin was a component of many flavin-enzyme co-groups and was involved in a wide range of redox reactions within cells (Ge et al., 2020). In the microbial metabolism during pickle fermentation, these flavin enzymes played a key catalytic role in the metabolism of carbohydrates, amino acids, and fats. They provided the material basis for microbial growth and the formation of pickle flavor substances.

### 3.5 Metabolic changes in pickles before and after fermentation

#### 3.5.1 Differential metabolites of pickles

**Fig. 5:**
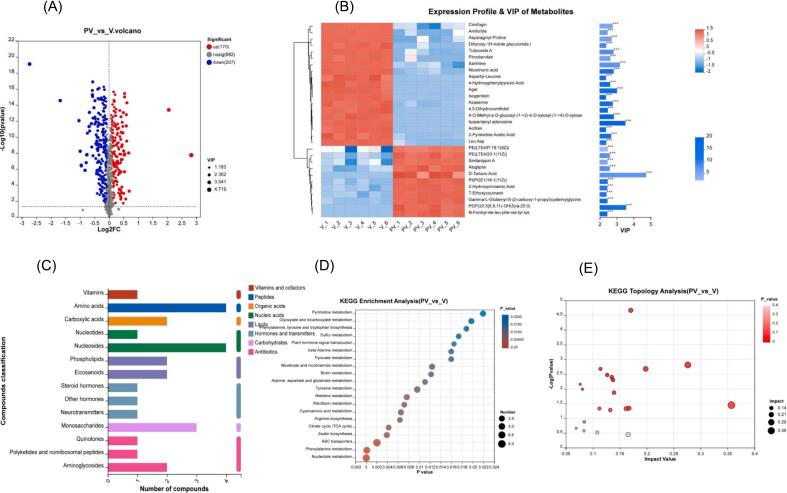
Comparison of metabolites and metabolic pathways of pickles before and after fermentation. (A) Volcanic maps. (B) VIP maps of differential metabolites. (C) Differential metabolite composition. (D-E) Enrichment of metabolic pathways during fermentation.

The variations in metabolite levels in pickles before and after fermentation were further examined. Based on the volcano plot (Fig. 5A), a total of 377 differential metabolites (*p* < 0.05, VIP > 1) were generated before and after pickle fermentation. These consisted of 170 up-regulated metabolites and 207 down-regulated metabolites, which could be classified into eight categories (Fig. 5C). The categories included five nucleic acids, four peptides, four antibiotics, four lipids, three hormones and transmitters, three carbohydrates, two organic acids, and one vitamin and co-factors.

Based on VIP values, the key differential metabolites produced during fermentation of pickles were analyzed (Fig. 5B). The analysis revealed that 19 metabolites such as amiloride, asparagine–proline, tuliposide A, pimobendan, xanthine, aspartyl–leucine and Leu-Asp were significantly down-regulated, which had certain similarities with the down-regulated metabolites of pickle solution. D-Tartaric acid, PE (LTE4/ P-18:1 (9Z)), N-Formyl-nle-leu-phe–nle–tyr–lys, Ginnalin B, alliin, valylproline, Gly-Asn-Phe, Val-Val-Tyr were significantly up-regulated.

Ginnalin B is a phenylpropanoid compound that usually appears in plants and has a variety of biological activities, including anti-hyperglycaemia, anti-oxidant, anti-inflammatory and anti-bacterial effects, as well as the prevention and treatment of diabetes(Honma et al., 2011). Ginnalin B may be formed or accumulate in pickles or fermented foods during curing and fermentation. The significant up-regulation of ginnalin B content (Fig. 4) in pickle solution following fermentation suggests that the metabolic activity of microorganisms may produce or enhance the content of this compound, which may offer health benefits for humans.

D-Glucitol, a commonly-known sugar alcohol also referred to as sorbitol, is typically utilized as a low-calorie sweetener in food products for diabetic patients. Additionally, D-Glucitol served as a laxative to stimulate intestinal peristalsis and assist in alleviating constipation (Valquiria & Ivone, 2013). The concentration of D-gluconol in the pickle solution increased significantly after fermentation. This indicated that an elevation in the D-gluconol content could prevent cardiovascular and cerebrovascular diseases, thereby being beneficial to human health.

Alliin, the primary organic sulfur compound in garlic, exhibited anti-diabetic, anti-cancer, antioxidant, and anti-inflammatory properties (Rodrigo et al., 2015). During the curing process, allicin was released from garlic into the pickles. This led to a significant increase (Fig. 4) in the concentration of allicin in the fermented pickle solution. As a result, both the flavor of the pickles and their health-promoting effects were enhanced.

Valyproline, a dipeptide composed of proline and valine, is a bioactive peptide. As reported by Liu & Wei (1981), it functions as an antioxidant and antibacterial agent, slows heart rate, and has anti-arrhythmic effects, making it a potential drug candidate. During the curing process, enzymes break down proteins into smaller peptide chains and amino acids. This led to a significant up-regulation of valyproline concentration in the fermented pickle solution (Fig. 4), which helps maintain the flavor and nutrition of pickles and extends their shelf-life. Short peptides such as Gly-Asn-Phe (glycine-asparagine-phenylalanine), Val-Val-Tyr (valine-valine-tyrosine), and Asn-Ile-Leu (aspartic acid-isoleucine-leucine) possess antioxidant and anti-inflammatory properties, enhance the body's immune system, and lower blood pressure. According to Joanna (2022), they can also act as neurotransmitters or regulators for signal transmission. These short peptides in pickled vegetables mainly originate from the decomposition of protein during fermentation. The significant up - regulation of oligopeptide concentration in the fermented pickle solution (Fig. 4K-L) not only enhances the flavor and taste of pickled vegetables but also endows them with high nutritional value.

#### 3.5.2 Key metabolic pathways of pickles

KEGG was used to annotate the differential metabolites before and after fermentation, explore the metabolic pathways involved in pickle fermentation (Fig. 5D) and select the top 20 metabolic pathways for analysis. Based on the *P*-values, nucleotide metabolism, phenylalanine metabolism and ABC transporter pathway were found to be the most significant metabolic pathways with the largest number of compounds. The topological analysis (Fig. 5E) showed that the biosynthesis and histidine metabolism pathways of keratin, subarginine and wax were the most significant and important metabolic pathways.

The nucleotide metabolic pathway, as described by Chandel (2021), is primarily engaged in the synthesis and degradation of nucleic acids and DNA. It encompassed a total of eight metabolites: deoxycytidine, hypoxanthine, deoxyuridine, adenosine, deoxyadenosine, xanthine, and uridine 5′-monophosphate. With the exception of adenosine and uridine 5′-monophosphate, all these metabolites were significantly down-regulated. Amino acid metabolic pathways, such as phenylalanine and histidine metabolism, are closely associated with the flavor of pickles. The phenylalanine metabolic pathway involved six metabolites: fumaric acid, phenylacetic acid, succinic acid, 2-hydroxycinnamic acid, and phenylacetylglutamine. In this pathway, phenylacetic acid, 2-hydroxycinnamic acid, and phenylacetylglutamine were significantly up-regulated, while fumaric acid, succinic acid, and other substances were significantly down-regulated. The histidine metabolic pathway involves four metabolites: histidine, L-histidine, imidazolinone-5-propionic acid, and 4-imidazolinone-5-propionic acid. All of these metabolites were significantly down-regulated.

The ABC transporter pathway is mainly associated with the nucleotide-binding domain and transmembrane domain (Hollenstein et al., 2007). It involved a total of eight metabolites, namely deoxycytidine, deoxyuridine, betaine, adenosine, l-glutamine, deoxyadenosine, and riboflavin. In this pathway, the levels of adenosine and mannitol were significantly up-regulated, whereas those of deoxycytidine, deoxyuridine, histidine, deoxyadenosine, and riboflavin were significantly down-regulated. The biosynthetic pathways of cutin, suberin, and wax were mainly responsible for synthesizing physical barrier and chemical antibacterial substances, as well as inducing resistance components. Among the metabolites involved, 9,10-dihydroxystearic acid and 16-hydroxycetanoic acid were significantly down-regulated. Mannitol was a polyol that played an important role in the response of microorganisms to environmental stress. In the pickle fermentation environment, where there were osmotic pressure changes, the accumulation of mannitol by microorganisms could regulate intracellular osmotic pressure, prevent cell water loss, and maintain the normal form and function of cells. Simultaneously, mannitol itself had a weak sweetness, which could improve the taste and flavor of pickles to a certain extent.

### 3.6 Source analysis of key metabolites in pickled vegetables

#### 3.6.1 Common and unique components of pickles and pickle solution

The common and unique components of metabolites in pickles and pickle solution were analyzed, focusing on the sources of beneficial and harmful metabolites. As shown in Fig. 2C, the Venn diagram clearly showed the similarities and differences of metabolites between the four groups. A total of 914 metabolites were identified in the pickle solution before and after fermentation (PVL\VL), including compounds such as pyroglutamyl glutamyl proline amide, ribavirin phosphate and elocalcitol. Additionally, 27 unique metabolites were found in the fermented pickle solution, including etiracetam, cohesive, histidine and allicin.

A total of 89 unique metabolites were identified, including emopamil and *N*-acetylcarbamate. There were 897 metabolites identified in pickles before and after fermentation (PV\V), including sunflower phenol creoside and thiomorph-3-carboxylate, etc. After fermentation, 22 unique metabolites of pickles were identified, including D-tartaric acid, 14,15-dichter and Knope. There were 853 metabolites in pickle solution and pickle (PVL\PV), including decyl alcohol, 12-hydroxy-dodecanoic acid and mannitol, etc. Additionally, 76 unique metabolites of pickled vegetables were identified, including D-tartaric acid and PE-NMe. Furthermore, 88 unique metabolites were detected in pickle solution, including 5-fluoruridine, yangonin and terbutaline. Harmful substances produced during the pickling process were present in pickled vegetables, but were not detected in the pickle solution.

#### 3.6.2 Source analysis of beneficial and harmful metabolites

It can be seen from Fig. 2D that metabolites such as keto-3-deoxyd-mannose-octotic acid, 3-denitrogenated adenosine, maltotriose, apigenin, n-ribose histidine, methionyllysine and S-lactoylglutathione were significantly different between vegetable and vegetable solution. After fermentation, the key differential metabolites found in pickle and pickle solution included legumin, cefixime, (+/−)-tryptophan, niacin ribonucleoside, keto-3-deoxyd-mannose-octanose acid and guanosine monophosphate.

Based on the cluster heat map depicting the different metabolite levels in raw vegetables, pickles, pickle solution, and vegetable solution (Fig. 3E), 32 types of metabolites from raw vegetables, such as indole-3-acetyl-*L*-phenylalanine, 4-vinylphenol sulfate, cis-aconite, aspartame, glyceraldehyde, 2,3,4,5-tetrahydro-2-pyridine carboxylic acid, and phenylpyruvate, were incorporated into the pickle solution, leading to a significant up-regulation of their levels. Conversely, 18 metabolites in the pickle solution, such as 9-octanecamide, oleic acid amide, palatinose, methylprednisone acetate, quercetin-3-O-xylosyl-glucoside, and l-glutamine, penetrated into the pickle.

## 4 Conclusion

In the pickled vegetables industry, low-salt fermented pickled vegetables are an inevitable trend, yet product quality remains inconsistent. Thus, exploring key metabolites and control targets in their fermentation process is crucial. This study explored the fermentation-induced quality changes in low-salt pickled vegetables, encompassing pH, texture, nitrite content, flavor, and metabolite composition. After identifying the primary flavor compounds in pickled vegetables, it analyzed the composition and potential origins of beneficial and harmful metabolites generated during fermentation. The study also uncovered the essence of substance transfer between pickled vegetables and their soaking solution. These findings offer valuable insights for the standardized production and quality enhancement of pickled vegetables. Notably, this study did not cover certain microorganisms in the fermentation of low-salt white radish. Future research will use multi-omics technology to explore the impact of microbial diversity and community composition on this process.

## CRediT authorship contribution statement

**Yanan Xia:** Writing – original draft, Software, Methodology, Data curation, Conceptualization. **Wenjing Zhu:** Software, Methodology. **Yufan Su:** Software, Methodology. **Yongfu Chen:** Writing – review & editing, Supervision.

## Declaration of competing interest

The authors declare that they have no known competing financial interests or personal relationships that could have appeared to influence the work reported in this paper.

## Data Availability

Data will be made available on request.

## Appendix A Supplementary data

Table. S1 Sensor Characteristics of E-nose Table.S2 Sensory scoring standard Image 1
