# Supplementary material for: Novel insights into the quality changes and metabolite transfer rules of pickles during fermentation: Pickle versus pickle solution
Source: Food Chem X. 2025 Jan 24;25:102203. doi: 10.1016/j.fochx.2025.102203 (PMC11838116; doi:10.1016/j.fochx.2025.102203)
Supplement: Supplementary file 1 — Table. S1 Sensor Characteristics of E-nose Table.S2 Sensory scoring standard [file mmc1.pdf]

Table. S1 Sensor Characteristics of E-nose

Sensors	Sensor characteristics	Response component
W1C(S1)	Sensitive to aromatic components	Methylbenzene(C_7H_8)
W5S(S2)	Sensitive to ammonia oxide	NO_2
W3C(S3)	Sensitive to aromatic components and ammonia	Benzene(C_6H_6)
W6S(S4)	Sensitive to hydrogen	H_2
W5C(S5)	Sensitive to aromatic components and alkanes	propane(C3H8)
W1S(S6)	Sensitive to methane	CH_4
W1W(S7)	Sensitive to organic and nitrifying components of hydrocarbons and sulfur	H_2S
W2S(S8)	Sensitive to ethanol	CO
W2W(S9)	Sensitive to organic sulfides and aromatic components	H_2S
W3S(S10)	Sensitive to alkanes	CH_4

Table.S2 Sensory scoring standard

Index	Evaluation criteria	Score range
Color(Score:25)	Normal color, luster, no stratification, clear soup	20~25
	Normal color, no luster	10~19
	Abnormal color, dull, black	0~10
Texture(Score:20)	Crisp, smooth taste, no rough feeling	15~20
	Crisp and tender, a little rough feeling	8~14
	Soft and rotten, obvious rough feeling	0~8
Taste(Score:20)	Palatability, with the unique taste of pickled radish, moderate saltiness and bitterness, no astringency , bitter aftertaste and astringency aftertaste	15~20
	A hint of bitterness, astringency, bitter and astringent aftertaste	8~14
	Too sour or too weak, has a raw taste of radish, bitter, astringent or rancidity, significant bitter and astringent aftertaste is , and has other undesirable odor	0~8
Preference degree(Score:35)	High (like)	30~35
	Moderate	20~29
	Low	0~19
